# Beta-lactamase induction and cell wall metabolism in Gram-negative bacteria

**DOI:** 10.3389/fmicb.2013.00128

**Published:** 2013-05-22

**Authors:** Ximin Zeng, Jun Lin

**Affiliations:** Department of Animal Science, The University of TennesseeKnoxville, TN, USA

**Keywords:** beta-lactamase, regulation, peptidoglycan

## Abstract

Production of beta-lactamases, the enzymes that degrade beta-lactam antibiotics, is the most widespread and threatening mechanism of antibiotic resistance. In the past, extensive research has focused on the structure, function, and ecology of beta-lactamases while limited efforts were placed on the regulatory mechanisms of beta-lactamases. Recently, increasing evidence demonstrate a direct link between beta-lactamase induction and cell wall metabolism in Gram-negative bacteria. Specifically, expression of beta-lactamase could be induced by the liberated murein fragments, such as muropeptides. This article summarizes current knowledge on cell wall metabolism, beta-lactam antibiotics, and beta-lactamases. In particular, we comprehensively reviewed recent studies on the beta-lactamase induction by muropeptides via two major molecular mechanisms (the AmpG–AmpR–AmpC pathway and BlrAB-like two-component regulatory system) in Gram-negative bacteria. The signaling pathways for beta-lactamase induction offer a broad array of promising targets for the discovery of new antibacterial drugs used for combination therapies. Therefore, to develop effective mitigation strategies against the widespread beta-lactam resistance, examination of the molecular basis of beta-lactamase induction by cell wall fragment is highly warranted.

## INTRODUCTION

Bacteria should continuously maintain and shape their envelopes to adapt enormous stresses they encounter in different niches and to meet physiological needs, such as growth and multiplication. Bacterial envelope is highly organized as a layer structure including cell wall, membrane(s), and the possible space between them. The structure of cell envelope varies in prokaryotes. In general, Gram-positive bacteria contain a thick layer of cell wall as well as a layer of cytoplasmic membrane. However, Gram-negative bacteria (e.g., *Escherichia coli*) typically contain an outer membrane, an intervening periplasmic space where a thin layer of cell wall resides, and a layer of cytoplasmic membrane.

The bacterial cell wall is unique to bacteria and plays a critical role in maintaining cell integrity. In addition, the conserved cell wall components, such as monomeric disaccharide tetrapeptide, could serve as a signal to trigger host immunologic or pathologic responses ( Goldman et al., 1982; Melly et al., 1984; Viala et al., 2004; Watanabe et al., 2004; Dziarski and Gupta, 2005; Cloud-Hansen et al., 2006; Strober et al., 2006). Thus, given its significant role in bacterial pathophysiology, cell wall has been an effective target for developing various antimicrobials with different mode of actions, such as beta-lactam and glycopeptide antibiotics. Of these, beta-lactam antibiotics are the most commercially available antibiotics in the market. Until 2010, beta-lactam antibiotics account for sales of approximately 53% of the total antibiotic market worldwide (42 billion US dollars; Hamad, 2010). Beta-lactam antibiotics inhibit bacterial cell wall biosynthesis, consequently leading to cell lysis and death. Specifically, beta-lactam antibiotics bind and acylate active site of penicillin-binding protein (PBP), the enzyme essential for the biosynthesis of bacteria cell wall.

To counteract bactericidal effect of beta-lactams, bacteria have quickly evolved defense systems in which production of beta-lactamase is a major beta-lactam resistance mechanism. Bacterial resistance to beta-lactam antibiotics has become a worldwide health care problem, as exemplified by the recent emergence of broad-range beta-lactam resistant NDM-1 (New Delhi metallo-beta-lactamase 1) strains ( Kumarasamy et al., 2010). Beta-lactamase is an enzyme that could hydrolyze beta-lactam ring, consequently deactivating beta-lactam antibiotics. In Gram-negative bacteria, the beta-lactamase was usually produced at very high concentration constitutively or by induction via direct interaction of beta-lactam antibiotic with regulatory system (e.g., MeBR1/MecI in *Staphylococcus aureus*; Kogut et al., 1956; Richmond, 1963, 1965; Pollock, 1965; Zhu et al., 1992; Fuda et al., 2005; Safo et al., 2005). In Gram-negative bacteria, the expression level of beta-lactamase is usually low; however, it has been observed that production of beta-lactamase was inducible but molecular basis for this phenomenon was not clear ( Ambler, 1980; Jacobs et al., 1997).

In the past, extensive research has focused on the structure, function, and ecology of beta-lactamases while limited efforts were placed on the regulatory mechanisms of beta-lactamases. In 1990s, the induction of beta-lactamase AmpC was observed to be correlated to the recycling process of cell wall in Gram-negative bacteria, which shed light on the molecular basis of beta-lactamase induction ( Jacobs et al., 1994). In the past two decades, accumulating evidence have shown the relationship between muropeptide release and beta-lactamase induction in Gram-negative bacteria ( Holtje et al., 1994; Jacobs et al., 1994, 1997; Korsak et al., 2005). However, in Gram-positive bacteria, there is little evidence showing the induction of beta-lactamases by liberated murein fragments. Recently, Amoroso et al. (2012) observed that a cell wall fragment could re-enter in the cytoplasm of *Bacillus licheniformis* and function as a signal to induce the expression of beta-lactamase. However, whether this cell wall fragment is the major signal for beta-lactamase induction in this Gram-positive bacterium still needs to be determined in the future. Given the lack of information on the relationship between beta-lactamase induction and cell wall metabolism in Gram-positive bacteria, in this review, we only summarize the relevant background information and recent research on the mechanisms of beta-lactamase induction by cell wall fragments in Gram-negative bacteria. In addition, we also discuss potential strategies to mitigate beta-lactam resistance by targeting beta-lactamase induction pathways.

## PEPTIDOGLYCAN BIOSYNTHESIS AND RECYCLING

**FIGURE 1:**
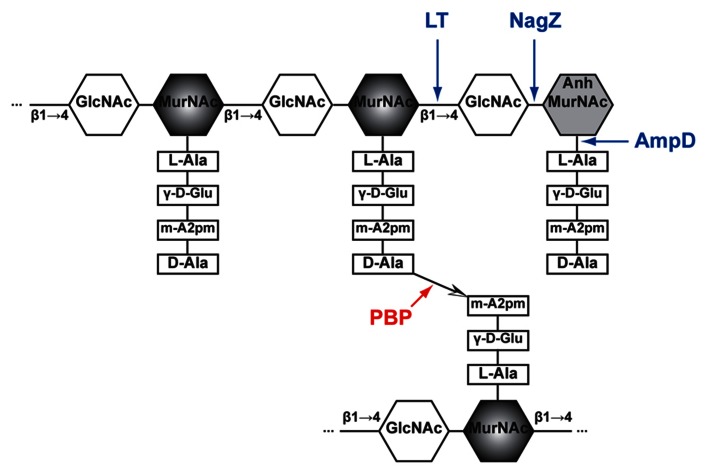
**Schematic structure of PG and target sites of different enzymes (pointed by color arrows).** The synthetic enzyme (PBP) is highlighted in red while the lytic enzymes (NagZ, AmpD, and LT) are highlighted in blue. Notably, NagZ and AmpD catalyze the liberated muropeptides instead of intact PG. Hexagons denote sugars while rectangles denote stem amino acids. The cross-linkage 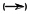 between the top and bottom glycan strands is D-Ala → meso-A2pm. LT, lytic transglycosylase; PBP, penicillin-binding protein, m-A2pm, meso-diaminopimelic acid; AnhMurNAc, 1,6-anhydro-MurNAc; β1 → 4, β-(1,4)-glycosidic bond.

In Gram-negative bacteria, peptidoglycan (PG), also called murein, is a mesh structure with units of continuous biopolymer residing on the intervening space between the outer and inner (cytoplasmic) membrane. Specifically, PG is a polysaccharide composed of repeating β-(1,4)-GlcNAc-β-(1,4)-MurNAc disaccharide interconnected by oligopeptide stems via covalent bond ( Glauner et al., 1988; **Figure 1**). The PG maintains cell integrity by sustaining internal osmotic pressure and keeps the regular bacterial shape. The glycan strand in *E. coli* is averagely composed of 29 disaccharide-peptide units ( Glauner, 1988).

The PG biosynthesis involves multi-stage enzymatic activities. First, the PG monomer unit (disaccharide with oligopeptide stem) is attached to a lipid in the cytoplasmic leaf of inner membrane ( van Heijenoort, 2001b; Barreteau et al., 2008; Bouhss et al., 2008). Second, the PG monomer-lipid intermediate is flipped into periplasm and catalyzed into the end of extending glycan chain by glycosyltransferases ( Goffin and Ghuysen, 1998; van Heijenoort, 2001a; Sauvage et al., 2008). Finally, the stem oligopeptides [L-Ala-γ-D-Glu-meso-A2pm-(L)-D-Ala-D-Ala pentapeptide in *E. coli*, **Figure 1**] that is linked to MurNAc are cross-linked to the adjacent stem oligopeptides from other glycan chains by transpeptidases ( Goffin and Ghuysen, 2002; Sauvage et al., 2008). These transpeptidases are the target of beta-lactam antibiotics and also called PBPs (including PBP1a, PBP1b, PBP1c, PBP2, and PBP3; Goffin and Ghuysen, 1998; Sauvage et al., 2008). Thus, PBPs are involved in the final stage of PG synthesis. Each bacterial cell may produce different PBPs, leading to various types of cross-linkage, such as D-Ala → (D-meso-A2pm, (L)-meso-A2pm → (D)-meso-A2pm, and so on ( van Heijenoort, 2011), for making a rigid mesh structure of PG.

Notably, PG is not a static biological structure. The structural units of PG changes dynamically during bacterial growth and doubling, with old units degraded and new materials added. Instead of starting over the complete *de novo* synthesis as described above, large quantities of the new materials added are recycled from the degraded PG units. It’s estimated that up to 60% of the parental cell wall is made of the recycled PG units during active bacterial growth ( de Pedro et al., 2001; Park and Uehara, 2008).

The PG recycling also involves multi-stage enzymatic activities. First, the lytic transglycosylase (LT) cleaves the glycan strand between the MurNAc and GlcNAc, and forms the 1,6-anhydro bond at the newly exposed MurNAc end in the mean time. With the aid of the endopeptidases (e.g., PBP4) that could break the cross-linkage between stem oligopeptides, anhydro muropeptide monomers (GlcNAc-anhydro-MurNAc-peptides) are liberated from PG. The main muropeptides are GlcNAc-anhMurNAc-L-Ala-γ-D-Glu-meso-A2pm-D-Ala (GlcNAc-anhydroMurNAc-tetrapeptide), with small amount of tri-, pentapeptides ( Glauner, 1988). Second, these muropeptides are transported into cytoplasm through the inner membrane transporter AmpG ( Park and Uehara, 2008). Subsequently, in cytoplasm, the GlcNAc sugar residue is removed by the glycoside hydrolase NagZ ( Cheng et al., 2000; Votsch and Templin, 2000). The resulting population of 1,6-anhydroMurNAc-oligopeptides are further transformed to UDP-MurNAc-pentapeptide ( Park and Uehara, 2008), a PG precursor that can be reincorporated into the PG biosynthesis pathway ( Park and Uehara, 2008). The muropeptides also could serve as a signal to induce the production of beta-lactamase, which will be discussed below in Section “Mechanisms of Beta-lactamase Induction.”

## BETA-LACTAM ANTIBIOTICS AND BETA-LACTAMASE

**FIGURE 2:**
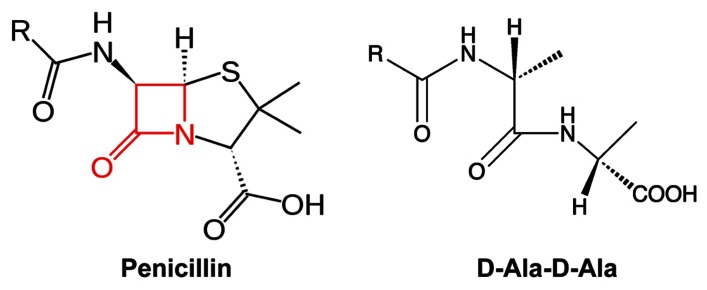
**The mimicry of beta-lactam antibiotics to D-alanyl-D-alanine (D-Ala-D-Ala).** The four-member lactam ring in penicillin was highlighted in red.

In 1928, Alexander Fleming observed the bactericidal effect of *Penicillium notatum*, leading to the identification of the first beta-lactam antibiotic, penicillin ( Fleming, 1929). Since then, a variety of beta-lactam antibiotics with different antimicrobial profiles have been discovered or synthesized, such as penicillin derivatives (penams), cephalosporins (cephems), monobactams, and carbapenems. All beta-lactam antibiotics share a common core containing a four-member beta-lactam ring (**Figure 2**). This beta-lactam ring displays phenomenal structural mimicry with the backbone of the D-alanyl-D-alanine, the substrate of PBP (**Figure 2**). Therefore, penicillin has been proposed to act as a substrate analog and binds to the active site of transpeptidases for inhibition of synthesis of the cross-linked PG ( Tipper and Strominger, 1965). This hypothesis was later supported by the evidence that transpeptidases could bind radioactive-labeled penicillin; thus, transpeptidases were also called as PBPs ( Cooper et al., 1949; Maass and Johnson, 1949a, 1949b; Cooper, 1955; Schepartz and Johnson, 1956; Markov et al., 1960; Spratt and Pardee, 1975).

Beta-lactam antibiotics have been a primary choice for physicians to treat bacterial infections due to their high specificity and potent killing effect. Clinical introduction of beta-lactam antibiotics has ever claimed to be a historical victory against bacterial infection; the mortality rate due to bacterial infections in the USA was drastically dropped from 797 to 36 per 100,000 individuals between 1900 and 1980 ( Armstrong et al., 1999). The emergence of antibiotic resistant bacteria quickly becomes the ghost of modern medicine ( Cohen, 2000). In fact, even during the ground-breaking discovery of penicillin, Alexander Fleming has already isolated the *E. coli*, *Salmonella enterica* serovar Typhi, and *Haemophilus influenza* strains that were resistant to penicillin ( Fleming, 1929). Although numerous efforts have been placed on the discovery new generation of beta-lactam antibiotics to further improve their clinical efficacy, bacteria have been evolving with an unbeatable pace to fail those new beta-lactams ( Culotta, 1994). To address this serious public health issue, it is imperative to study the molecular basis of beta-lactam resistance so that we can overcome beta-lactam resistance by targeting resistance mechanisms.

The molecular mechanisms of beta-lactam resistance have been widely studied ( Ogawara, 1981; Fuda et al., 2004; Jovetic et al., 2010; Harris and Ferguson, 2012). To evade the bactericidal effects of beta-lactam antibiotics, Gram-negative bacteria have evolved multiple strategies, such as production of beta-lactamases ( Korfmann and Wiedemann, 1988; Jacoby, 2009), production of novel PBPs with reduced affinity to beta-lactam antibiotics ( Fuda et al., 2004), reducing beta-lactam antibiotics entry through mutations in porins, and expelling beta-lactam antibiotics out of cells using multi-drug efflux pumps ( Kohler et al., 1999). Of these mechanisms, producing beta-lactamases, the enzymes that could hydrolyze beta-lactam ring, is still the most efficient strategy ( Abraham and Chain, 1940; Jacoby and Munoz-Price, 2005). It has been proposed that beta-lactamases and the PBPs may share a common ancestor due to the presence of certain sequence homology ( Massova and Mobashery, 1998). Recently, Fernandez et al. (2012) observed that overexpression beta-lactamases changed the PG composition and affected bacterial fitness, likely due to the residual transpeptidase activity of the beta-lactamases.

Given the tight link between beta-lactam resistance and the beta-lactamase activity, it is not surprising that past studies were primarily focused on the structure, function, and ecology of beta-lactamases. Particularly, many epidemiological, clinical, and ecological studies are focused on the detection and characterization of specific beta-lactamase genes with little attention on the regulatory mechanism of beta-lactamases. The first “cryptic” beta-lactamase, AmpC (originally named AmpA), was identified in beta-lactam sensitive *E. coli* K-12 by stepwise selection on beta-lactam antibiotics containing medium ( Eriksson-Grennberg et al., 1965; Eriksson-Grennberg, 1968). The beta-lactam resistant derivatives constitutively produced high-level of beta-lactamases, suggesting the presence of an inducible beta-lactamase gene in *E. coli* K-12 ( Linstrom et al., 1970). Later, the AmpC gene was cloned and characterized as a beta-lactamase ( Jaurin and Grundstrom, 1981). The expression of *ampC* normally is maintained at low level and dependent on growth rate ( Jaurin et al., 1981). However, a single nucleotide mutation in the promoter region (likely an attenuator) of *ampC* led to overexpression of beta-lactamase, indicating that the *ampC* was subjected to regulation ( Jaurin et al., 1981). Then the *ampC* was observed to be widely distributed in different enterobacterial species, such as *Salmonella enterica* serovar Typhimurium, *Pseudomonas aeruginosa*, *Serratia marcescens*, and *Klebsiella pneumonia*; interestingly, the *ampC* was inducible under treatment of beta-lactam antibiotics ( Bergstrom et al., 1982). However, the expression of *ampC* in *E. coli* was not induced by beta-lactam antibiotics due to the lack of a regulator gene *ampR* adjacent to the *ampC* in the chromosome ( Honore et al., 1986). Complementation of *E. coli* with a plasmid containing the *ampR–ampC* operon from *Enterobacter cloacae* restored the phenotype of beta-lactamase induction ( Kraft et al., 1999).

The induction of beta-lactamase is of great clinical importance. For example, prolonged administration of beta-lactam antibiotics could lead to emergence of *P. aeruginosa* mutants resistance to multiple beta-lactam antibiotics, eventually leading to treatment failure and patient death ( Livermore, 1987; Sanders, 1987; Giwercman et al., 1990; Juan et al., 2005). Therefore, significant progresses have been made on the molecular basis of the beta-lactamase induction in Gram-negative bacteria in the past two decades.

## MECHANISMS OF BETA-LACTAMASE INDUCTION

**FIGURE 3:**
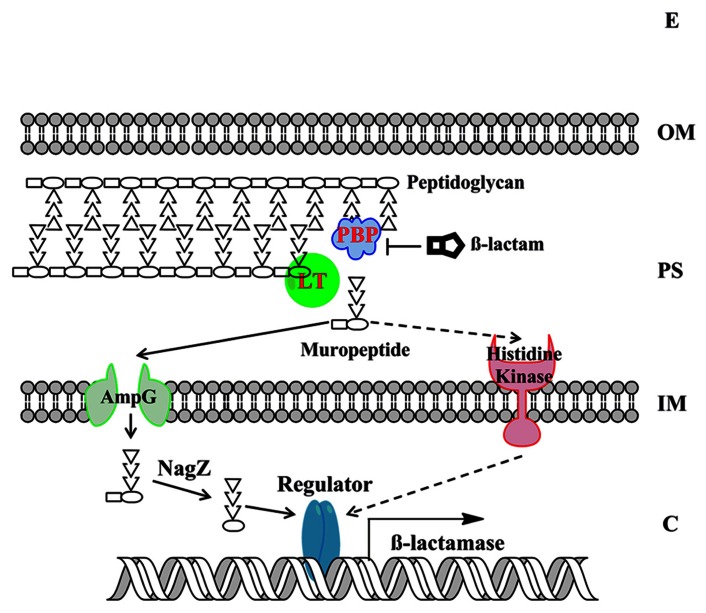
**The model of beta-lactamase induction in Gram-negative bacteria.** The beta-lactamase induction by muropeptides via two major molecular mechanisms, the AmpG–AmpR–AmpC pathway and the BlrAB-like two-component regulatory system, are presented. The signaling pathway via two-component regulatory system is only supported by limited studies to date and is shown in dashed arrows. The “Regulator” denotes AmpR-like regulator or two-component response regulator. The “beta-lactamase” denotes the beta-lactamase that is subjected to induction. E, extracellular environment; OM, outer membrane; PS, periplasmic space; IM, inner membrane; C, cytoplasm.

Understanding the molecular basis of beta-lactamase induction would facilitate us to develop effective combination therapy strategy by inhibiting the induction of beta-lactamase. Gram-negative bacteria have evolved two major mechanisms for beta-lactamase induction, the AmpG–AmpR–AmpC pathway and the two-component regulatory system (TCRS; **Figure 3**). Recent progresses in this significant research area are summarized below.

### THE AmpG–AmpR–AmpC PATHWAY

As mentioned above, in many bacteria belonging to Enterobacteriaceae family, AmpC expression is induced by beta-lactam antibiotics. Since beta-lactam antibiotics treatment can trigger the release of large amount of muropeptides in periplasm, which could be subjected to cell wall recycling process, the relationship between cell wall recycling and beta-lactamase induction has been examined and confirmed in recent studies. Briefly, in the AmpG–AmpR–AmpC pathway, beta-lactam antibiotics treatment breaks the balance of PG biosynthesis (e.g., due to the inhibited PBP and the functional LT), consequently liberating GlcNAc-anhydro-MurNAc-oligopeptides in periplasm ( Templin et al., 1992). The GlcNAc-anhydro-MurNAc-oligopeptides are further transported into cytoplasm through AmpG transporter ( Park and Uehara, 2008). The GlcNAc moiety is removed by enzyme NagZ, leading the accumulated PG products (mainly anhydro-MurNAc-tetrapeptides). In cytoplasm, anhydro-MurNAc-oligopeptide are the inducer of beta-lactamase expression through the interaction with AmpR ( Lindquist et al., 1989; Jacobs et al., 1997).

AmpR is a LysR type transcriptional regulator and is encoded immediately upstream of *ampC* with opposite direction ( Lindquist et al., 1989; Jacobs et al., 1997). AmpR was demonstrated as an activator for *ampC* using *in vitro* transcription assay ( Jacobs et al., 1997). However, production of *ampC* was still repressed even if bacterial host contains functional AmpR, unless exogenous beta-lactam antibiotic was added ( Honore et al., 1986; Lindquist et al., 1989; Lodge et al., 1990; Jacobs et al., 1997). Therefore, it has been hypothesized that the activator function of AmpR was inhibited by certain cellular metabolite, which was demonstrated as the cell wall synthesis precursor, UDP-MurNAc-pentapeptide ( Jacobs et al., 1997). This inhibition was abolished in the mutant with point mutation in AmpR (G102E; Bartowsky and Normark, 1991), indicating the role of the residue G for the association of UDP-MurNAc-pentapeptide. Upon the treatment of beta-lactam antibiotics, the accumulated intracellular anhydro-MurNAc-oligopeptides could displace the AmpR-associated UDP-MurNAc-pentapeptide, triggering conformational change of AmpR, and subsequently activating the transcription of *ampC* ( Jacobs et al., 1997). The DNase I-protection assay showed the binding site of AmpR was in a 39-bp region upstream of the *ampC* transcription start site (-40 to -88; Jacobs et al., 1997). Interestingly, AmpR in *P. aeruginosa* is a global transcriptional factor whose regulon includes beta-lactamases, proteases, quorum sensing, and other virulence factors ( Kong et al., 2005; Balasubramanian et al., 2012).

Among the PG cycling process, there is a negative effector to fine-tune the expression of AmpC. A cytoplasmic *N*-acetylmuramoyl-L-alanine amidase, named AmpD ( Holtje et al., 1994), could dissociate stem peptides from the anhydro-MurNAc or GlcNAc-anhydro-MurNAc, therefore, reducing concentrations of the inducing muropeptides and mitigating the overexpression of AmpC ( Jacobs et al., 1994).

Consistent with these observations on the relationship between PG cycling and beta-lactamase induction, perturbation of PG recycling also affected AmpC induction, suggesting potential pharmaceutical targets. For example, overproduction of the LT MltB stimulated beta-lactamase induction whereas specific inhibition of LT Slt70 by bulgecin repressed AmpC expression ( Kraft et al., 1999). In addition, mutation of all six LT enzymes (Slt70, MltA, MltB, MltC, MltD, and EmtA) in *E. coli* decreased the beta-lactamase activities ( Korsak et al., 2005).

Different versions of AmpG–AmpR–AmpC regulatory pathways exist in bacteria. For example, *E. coli* and *Shigella* spp. lacks an *ampR* gene ( Bergstrom et al., 1982; Honore et al., 1986), leading to the low level, non-inducible expression of AmpC. The AmpC gene in *E. coli* was primarily regulated by an attenuator sequence in promoter region ( Jaurin et al., 1981). The overexpression of AmpC can be achieved either by mutating attenuator ( Jaurin et al., 1981) or by introducing an AmpR regulator ( Kraft et al., 1999); the similar pathway was also observed in *Acinetobacter baumannii* ( Bou and Martinez-Beltran, 2000). In *Salmonella*, the chromosomal AmpC–AmpR is usually absent, which may be due to unbearable production cost of AmpC ( Morosini et al., 2000). However, clinical *Salmonella* strains can acquire AmpC–AmpR through horizontally transferred mobile elements ( Barnaud et al., 1998). In *Serratia marcescens*, besides AmpR regulation, the post-transcriptional regulation also influences the expression of AmpC. Specifically, the half-life of *ampC* transcript could be affected by a 126-bp, non-encoding region that forms a stem-loop structure ( Mahlen et al., 2003). In *P. aeruginosa* PAO1, interestingly, there are three copies of *ampD* genes, which contributed to the stepwise up-regulation of AmpC with the discrete mutation of each copy of *ampD* ( Juan et al., 2006).

### THE BlrAB-LIKE TWO-COMPONENT REGULATORY SYSTEM

The TCRS, which involves sensing specific environmental stimuli ( Capra and Laub, 2012), was also observed to be involved in the induction of beta-lactamase. In *Aeromonas* spp., the AmpC and two other chromosomally encoded beta-lactamases were regulated by the response regulator BlrA of a TCRS instead of an AmpR-type regulator ( Alksne and Rasmussen, 1997). Complementation study demonstrated that overexpression of BlrA in *E. coli* enhanced the expression of the *Aeromonas*-derived beta-lactamase in *E. coli* MC1061 while the beta-lactamase was expressed at low level in the absence of BlrA ( Alksne and Rasmussen, 1997).

The closest TCRS homolog of BlrAB in *E. coli* is CreBC ( Amemura et al., 1986; Wanner and Wilmes-Riesenberg, 1992). Interestingly, the beta-lactamases from *Aeromonas hydrophila* could be regulated by the CreBC TCRS system in the Cre^+^ *E. coli* strain such as DH5α ( Avison et al., 2000, 2001). The “*cre*/*blr*-tag” signature, which is the “TTCACnnnnnnTTCAC” motif located in the promoter of Cre-regulon, was identified in *E. coli* ( Avison et al., 2001). These “cre/blr-tag” also reside in promoters of *Aeromonas*-derivative beta-lactamases ( Niumsup et al., 2003), and the induction of those beta-lactamases by overexpressed BlrA was dependent on the presence of “*cre/blr*-tag” ( Avison et al., 2004). In *P. aeruginosa*, inactivation of a non-essential PBP was shown to trigger overproduction of a chromosomal AmpC gene and this overproduction is dependent on CreBC TCRS ( Moya et al., 2009). Interestingly, among the 32 tested *E. coli* TCRS response regulators, overexpression of FimZ conferred increased level of beta-lactam resistance through the action of AmpC in *E. coli* ( Hirakawa et al., 2003).

Despite above evidence showing that TCRS is also involved in the induction of beta-lactamase, the identity of the corresponding cues to which the TCRS respond for beta-lactamase induction is still unknown. We speculate that specific degraded PG components may serve as a signal for the response regulator to induce the production of beta-lactamase. This hypothesis needs to be examined in the future.

### OTHER MECHANISMS

Another novel beta-lactamase induction pathway was discovered in *Ralstonia pickettii* ( Girlich et al., 2006). The chromosomally encoded beta-lactamases (OXA-22 and OXA-60) were regulated by ORF-RP3 (short for RP3), a gene located at 192-bp upstream of the ATG codon of *oxa-60*. Inactivation of RP3 resulted in the abolishment of induction of the both beta-lactamases; complementation of the RP3 restored the inducible expression of OXA-22 and OXA-60 ( Girlich et al., 2006). DNase I footprinting showed that RP3 specifically bound to tandem repeats upstream at the transcriptional start sites of OXA-22 and OXA-60 genes, suggesting RP3 is a novel positive-regulator for beta-lactamase induction ( Girlich et al., 2009).

## PHARMACEUTICAL IMPLICATIONS OF BETA-LACTAMASE INDUCTION MECHANISM

Discovery of beta-lactamase inhibitors is a promising strategy to combat the prevalent beta-lactam resistance ( Bush and Macielag, 2010; Harris and Ferguson, 2012). However, this approach is challenged by the variable affinity of the inhibitors to different beta-lactamases and by the overwhelming quantity of the beta-lactamases produced in resistant cells. Based on the information reviewed here, we propose that the signaling pathways of beta-lactamase induction offer a broad array of promising targets for the discovery of new antibacterial drugs used for combination therapies. The inhibitors targeting beta-lactamase induction pathway may prevent the emergence of beta-lactam resistance and enhance the efficacy of clinical beta-lactam antibiotics, as what we have observed for the efflux pump inhibitors ( Lomovskaya and Bostian, 2006). In supporting this hypothesis, the frequency of emergence of ceftazidime resistance in *blrAB* mutant in *P. aeruginosa* was below the detection limit (<1 × 10^-^^11^), which is far below that for the wild-type parent strain (3 × 10^-^^8^; Moya et al., 2009).

The potential targets in the beta-lactamase induction pathway as well as the known inhibitors are summarized in **Table 1**. Several inhibitors have been identified for LTs that play a critical role in the initializing the PG cycling. The LT inhibitor bulgecin could induce cell lysis and morphology changes in the presence of beta-lactam antibiotics although bulgecin alone did not show any antibacterial activity against *E. coli* ( Imada et al., 1982; Nakao et al., 1986; Bonis et al., 2012). The major molecular target of bulgecin was the soluble LT Slt70 ( Templin et al., 1992). In a 2.8-Å resolution crystallographic structure of Slt70-bulgecin complex, one single bulgecin molecule was found to be located in the active site of Slt70, indicating that bulgecin may act as an analog of an oxocarbenium ion intermediate in the reaction catalyzed by Slt70 ( Thunnissen et al., 1995). The beta-hexosaminidase inhibitor *N*-acetylglucosamine thiazoline (NAG-thiazoline) was also found to inhibit the LT sMltB from *P. aeruginosa* ( Reid et al., 2004a, b). Another inhibitor, hexa-*N*-acetylchitohexaose, can inhibit the LT from bacteriophage lambda ( Leung et al., 2001). Interestingly, a proteinaceous inhibitor of vertebrate lysozymes (Ivy), which has conserved CKPHDC motif, was also found to control the autolytic activity of bacterial LTs ( Clarke et al., 2010).

**Table 1:** The inhibitors targeting the beta-lactamase induction pathway.

**Target**	**Function**	**Inhibitor**
LT	Non-hydrolytic cleave PG with the concomitant formation of 1,6-anhydro-MurNAc	Bulgecin A (Templin et al., 1992); NAG-thiazoline ( Reid et al., 2004a, b); hexa-*N*-acetylchitohexaose (Leung et al., 2001); Ivy (Clarke et al., 2010)^a^
NagZ	Cleave disaccharide oligopeptides to release 1,6-anhydro-MurNAc-peptide	PUGNAc, EtBuPUG (Stubbs et al., 2007)
AmpG	Inner membrane permease of the 1,6-GlcNAc-anhydro-MurNAc-peptides	CCCP (Zhang et al., 2010)
AmpR	Binary regulator of AmpC	UDP-*N*-acetylmuramic acid peptides (Jacobs et al., 1997)

*^a^Proteinaceous inhibitor, also the inhibitor of vertebrate lysozymes.*

Regarding other targets in beta-lactamase induction pathway, PUGNAc and modified EtBuPUG can inhibit the function of NagZ by the mimicry of the oxocarbenium ion-like transition state ( Stubbs et al., 2007). Unlike PUGNAc that is also a potent inhibitor against human *O*-GlcNAcase and beta-hexosaminidase, EtBuPUG displayed 100-fold selectivity toward to NagZ. The function of inner membrane permease AmpG in laboratory strains of *P. aeruginosa* can be inhibited by carbonyl cyanide *m*-chlorophenylhydrazone (CCCP), a general inhibitor of proton motive force, consequently leading to an increased susceptibility to beta-lactam antibiotics ( Cheng and Park, 2002; Zhang et al., 2010). However, it is important to mention that CCCP also targets other energy-dependent systems, such as drug efflux pump; thus, the linkage between reduced beta-lactam resistance and AmpG inhibition was not clearly demonstrated in these studies.

Although a panel of inhibitors that target the PG recycling pathway have been identified (**Table 1**), it is still largely unknown if these inhibitors repress the inducible beta-lactam resistance effectively in Gram-negative bacteria, consequently enhancing the efficacy of clinical beta-lactam antibiotics. This knowledge gap needs to be filled in the future. In addition, similar to all infectious disease drug developments, discovery of a promising inhibitor targeting the beta-lactamase induction pathway and conversion such inhibitor into a clinically useful therapeutic agent are likely a lengthy and challenging process. Some key issues, such as toxicity, stability, bioavailability, and production cost, must be addressed. Despite these challenges, it is imperative to develop clinically useful inhibitors to suppress beta-lactamase induction and enhance “shelf-life” of a broad spectrum of beta-lactam antibiotics against bacterial pathogens. To achieve this goal, in-depth structural and functional studies are needed for the potential targets (**Table 1**), which is critical for identifying corresponding inhibitors using various modern approaches, such as high-throughput screening of chemical compound library, homology modeling and molecular docking.

## Conflict of Interest Statement

The authors declare that the research was conducted in the absence of any commercial or financial relationships that could be construed as a potential conflict of interest.

## Abbreviations

EPR: electron paramagnetic resonance
FTIR: Fourier transform infrared
OEC: oxygen-evolving complex
OTG: octyl-β-D-thioglucopyranoside
PSII: photosystem II
Q_A_: primary quinone electron acceptor in PSII
XRD: X-ray diffraction
